# Impact of R-Carvedilol on β2-Adrenergic Receptor-Mediated Spontaneous Calcium Release in Human Atrial Myocytes

**DOI:** 10.3390/biomedicines10071759

**Published:** 2022-07-21

**Authors:** Sergi Casabella-Ramón, Verónica Jiménez-Sábado, Carmen Tarifa, Sandra Casellas, Tien Tina Lu, Paloma Izquierdo-Castro, Ignasi Gich, Marcel Jiménez, Antonino Ginel, José M. Guerra, S. R. Wayne Chen, Raul Benítez, Leif Hove-Madsen

**Affiliations:** 1 Instituto de Investigaciones Biomédicas de Barcelona, CSIC, 08036 Barcelona, Spain; scasabella@santpau.cat (S.C.-R.); carmen.tarifa@cnic.es (C.T.); paloma.izquierdo@iibb.csic.es (P.I.-C.); 2 IIB Sant Pau, Hospital de la Santa Creu i Sant Pau, 08041 Barcelona, Spain; vjimenezs@santpau.cat (V.J.-S.); igich@santpau.cat (I.G.); jguerra@santpau.cat (J.M.G.); 3 Department of Cell Biology, Physiology and Immunology and Neuroscience Institute, Universitat Autònoma de Barcelona, 08193 Barcelona, Spain; mjimenez@uab.cat; 4 CIBER Enfermedades Cardiovasculares (CIBERCV), Instituto de Salud Carlos III, 28029 Madrid, Spain; 5 Servicio de Cirugía Cardíaca, Hospital de la Santa Creu i Sant Pau, 08025 Barcelona, Spain; scasellas@santpau.cat (S.C.); aginel@santpau.cat (A.G.); 6 Faculty of Medicine, University of British Columbia, Vancouver, BC V6T 1Z4, Canada; tina7lu@gmail.com; 7 CIBER Epidemiología y Salud Pública (CIBERESP), Instituto de Salud Carlos III, 28029 Madrid, Spain; 8 CIBER Enfermedades Hepáticas y Digestivas (CIBEREHD), Instituto de Salud Carlos III, 28029 Madrid, Spain; 9 Servicio de Cardiología, Hospital de la Santa Creu i Sant Pau, Universitat Autònoma de Barcelona, 08025 Barcelona, Spain; 10 Department of Physiology and Pharmacology, Libin Cardiovascular Institute of Alberta, University of Calgary, Calgary, AB T2N 2T9, Canada; swchen@ucalgary.ca; 11 Departament d’Enginyeria de Sistemes, Automàtica i Informàtica Industrial, Universitat Politècnica de Catalunya, 08034 Barcelona, Spain; raul.benitez@upc.edu

**Keywords:** β-adrenergic receptor blocker, human atrial myocyte, sarcoplasmic reticulum, calcium spark, arrhythmia, carvedilol

## Abstract

A hallmark of atrial fibrillation is an excess of spontaneous calcium release events, which can be mimicked by β1- or β2-adrenergic stimulation. Because β1-adrenergic receptor blockers (β1-blockers) are primarily used in clinical practice, we here examined the impact of β2-adrenergic stimulation on spontaneous calcium release and assessed whether the R- and S-enantiomers of the non-selective β- blocker carvedilol could reverse these effects. For this purpose, human atrial myocytes were isolated from patients undergoing cardiovascular surgery and subjected to confocal calcium imaging or immunofluorescent labeling of the ryanodine receptor (RyR2). Interestingly, the β2-adrenergic agonist fenoterol increased the incidence of calcium sparks and waves to levels observed with the non-specific β-adrenergic agonist isoproterenol. Moreover, fenoterol increased both the amplitude and duration of the sparks, facilitating their fusion into calcium waves. Subsequent application of the non β-blocking R-Carvedilol enantiomer reversed these effects of fenoterol in a dose-dependent manner. R-Carvedilol also reversed the fenoterol-induced phosphorylation of the RyR2 at Ser-2808 dose-dependently, and 1 µM of either R- or S-Carvedilol fully reversed the effect of fenoterol. Together, these findings demonstrate that β2-adrenergic stimulation alone stimulates RyR2 phosphorylation at Ser-2808 and spontaneous calcium release maximally, and points to carvedilol as a tool to attenuate the pathological activation of β2-receptors.

## 1. Introduction

Increased sympathetic nerve density has been observed in atrial samples from patients with atrial fibrillation (AF) [1,2]. The sympathetic activity in the atrium is mainly the result of the activation of G-protein-coupled β-adrenergic receptors [3,4]. This initiates an intracellular signaling cascade that involves the activation of adenylyl cyclase and leads to increased cyclic AMP (cAMP) levels and protein kinase A (PKA) activation [3,4,5]. This, in turn, induces phosphorylation of a number of calcium regulatory proteins, including the cardiac ryanodine receptor (RyR2) [6,7], that favors spontaneous calcium release from the sarcoplasmic reticulum (SR) and the induction of atrial arrhythmias [3,8,9,10,11].

The functional impact of β-adrenergic stimulation has been extensively studied in both atrial and ventricular myocytes, but most functional studies have addressed the overall response to non-selective β-adrenergic agonists such as isoproterenol (Iso). Recently, we have shown that β2-adrenergic stimulation of mouse cardiomyocytes mimics the alterations in intracellular calcium homeostasis observed in patients with atrial fibrillation [12]. In clinical practice, a variety of β-adrenergic receptor blockers (β-blockers) are used, among others, to prevent cardiac arrhythmia. Among these, carvedilol stands out because it inhibits β2-adrenergic receptors more strongly than β1-adrenergic receptors [13], and also because it modifies RyR2 gating directly [14]. Some studies have shown that carvedilol suppresses RyR2-mediated calcium waves and prevents calcium release-induced triggered ventricular arrhythmias in patients with catecholaminergic polymorphic ventricular tachycardia (CPVT) or heart failure (HF) [14,15]. Unfortunately, the carvedilol concentration necessary to modify RyR2 gating may also produce adverse effects caused by excessive β-blockade, such as bradycardia and hypotension [16,17]. However, carvedilol is currently used as a racemic mixture of the R- and S-enantiomers [18,19,20] that have differential effects on β-receptors and RyR2 activity. Thus, the R-Carvedilol (R-Carv) enantiomer does not appear to have β-blocking activity [18,20], but Zhang et al. [21] demonstrated that it suppresses ventricular tachycardia in mice with the CPVT-causing RyR2 mutation (R4496C) by directly modifying RyR2 gating without lowering the heart rate or blood pressure.

Because atrial fibrillation has been associated with an elevated incidence of spontaneous calcium release events that concur with increased RyR2 phosphorylation, and β2-adrenergic stimulation mimics these effects [12], the present study aims to determine the impact of β2-adrenergic stimulation on calcium homeostasis in human atrial myocytes and compare it to stimulation with a non-selective agonist such as Iso. Furthermore, the study aims to compare the ability of the R- and S-Carvedilol (S-Carv) enantiomers to reverse the effects of β2-adrenergic stimulation on spontaneous calcium release.

## 2. Materials and Methods

### 2.1. Study Population

We analyzed 37 consecutive patients undergoing cardiac surgery in our institution. All patients gave written consent to obtain the right atrial tissue samples that would otherwise have been discarded during the surgical intervention. The study protocol was approved by the Ethics Committee at Hospital de la Santa Creu i Sant Pau (Spain). Clinical characteristics, echocardiographic data, and pharmacologic treatments of the patients are summarized in Table 1.

### 2.2. Study Protocol

Right atrial myocardial samples were obtained prior to the cannulation of the right atrial appendage in surgery requiring extracorporeal circulation. Excised tissue samples were immediately stored in cold oxygenated calcium-free Tyrode solution containing 30 mM butenodione monoxime. Once in the laboratory, the tissue was snap-frozen or used for myocyte isolation within 5–10 min after excision. For myocyte isolation, the tissue was cleaned and cut into small pieces (1 mm × 1 mm) that were digested in a Ca^2+^-free Tyrode solution with 1.4 mg/mL collagenase (Worthington Type 4, 275 units/mg), 0.2 mg/mL proteinase (Sigma type XXIV, 11 u/mg solid), and 2 mg/mL bovine fatty acid-free serum albumin (BSA) and incubated at 37 °C for 30 min. After 30 min, cells were dissociated from the tissue fragments using a Pasteur pipette, and suspended in Ca^2+^-free Tyrode solution with 10 mg/mL BSA. The remaining tissue was digested in fresh enzyme solution containing 0.8 mg/mL collagenase and 2 mg/mL BSA at 37 °C for 15 min. This process was repeated 3 or 4 times. Subsequently, Ca^2+^ was gradually increased to 600 µM in the Ca^2+^-free solution containing the isolated cells. Only elongated cells with clear cross striations and without abnormal granulation were used for experiments. Depending on the yield of the myocyte isolation, cells were used for one or several experimental protocols, and, therefore, the total number of patients was larger than the number of patients in a specific experimental protocol. Usually, calcium imaging experiments were performed in 2–4 cells per patient and immunofluorescent labeling experiments in 3–5 cells per patient. The number of cells and patients are given in the figure legends as (number of cells/number of patients).

### 2.3. In Vitro Drug Testing

Isolated myocytes were exposed to R-Carv or S-Carv to explore the effects of the two enantiomers on RyR2 phosphorylation, calcium sparks, and calcium waves induced by β2-adrenergic stimulation with the β2-agonist Feno (3 µM).

### 2.4. Immunofluorescent Labeling

Isolated myocytes were subjected to immunofluorescent labeling as previously described [22]. Briefly, myocytes were fixed with 5% paraformaldehyde for 10 min at room temperature. Subsequently, cells were incubated with PBS/Glycine 0.1 M during 10 min and thereafter with PBS/0.2% Triton X-100 for 15 min to permeabilize the cells. To block the non-specific sites, the cells were incubated with PBS/0.2% Tween 20, and 10% horse serum, for at least 30 min. Total and Ser-2808 phosphorylated RyR2 were labeled using the primary antibodies mouse anti-RyR2 (1:1200; C3-33 NR07, Calbiochem, San Diego, CA, USA) and rabbit anti-Ser-2808P (1:1200; A010-30, Badrilla, Leeds, UK). Antibodies AlexaFluor 488 anti-mouse (1:2000; A21200, Molecular Probes, Eugene, OR, USA) and AlexaFluor 594 anti-rabbit (1:1500; A11012, Molecular Probes, Eugene, OR, USA) were used to stain total RyR2 green and Ser-2808 phosphorylated RyR2 red. Images were acquired with a confocal microscope (Leica AOBS SP5, Wetzlar, Germany) and a 63× glycerol immersion objective. To determine the ability of R-Carv to attenuate fenoterol (Feno)-induced RyR2 phosphorylation, data were fit with a Hill equation in order to obtain the R-Carv concentration required to achieve a half-maximal reduction in the response to Feno.

### 2.5. Confocal Calcium Imaging

To visualize changes in the intracellular calcium concentration, myocytes were loaded with 2 µM CAL-520 AM for 20 min at room temperature, followed by washing and de-esterification for at least 30 min. Confocal calcium images (512 × 140 pixels) were recorded at 90 Hz using a resonance-scanning confocal microscope (Leica SP5 AOBS, Wetzlar, Germany) with a 63x glycerol immersion objective in the frame-scanning mode. CAL-520 was excited at 488 nm and emission was measured between 500 and 650 nm with a Leica Hybrid Detector. Laser power was set to 20% of 100mW and then attenuated to 4%. Experiments were performed at room temperature and calcium sparks were detected using custom-made algorithms implemented using MATLAB (Mathworks Inc., Boston, MA, USA) as previously described [23].

### 2.6. Data Analysis

Electrophysiological and molecular biological analyses were performed without knowledge about clinical data and, unless otherwise stated, values for quantitative variables were averaged for each patient and given as mean ± s.e.m. Clinicians analyzing the medical records had no access to the experimental results.

### 2.7. Statistical Analysis

For calcium spark analysis, results are represented in bar graphs with mean ± s.e.m. and individual data points. For normally distributed quantitative variables, statistical significance was evaluated using *t*-test (for pairs) or analysis of variance (ANOVA). For variables with clear asymmetry, statistical significance was evaluated using Wilcoxon’s rank sum test (for pairs), Kruskal–Wallis test or ANOVA test with Welch correction. The specific statistical test is indicated in text or figure legends. Analyses were performed using IBM SPSS Statistics for Windows (V26.0) or RStudio 1.4.1717. Statistically significant effects are indicated with *p*-values or *: *p* < 0.05, **: *p* < 0.01; ***: *p* < 0.001.

## 3. Results

### 3.1. Impact of β2-Adrenergic Stimulation on Intracellular Calcium Homeostasis in Human Atrial Myocytes

**Figure 1:**
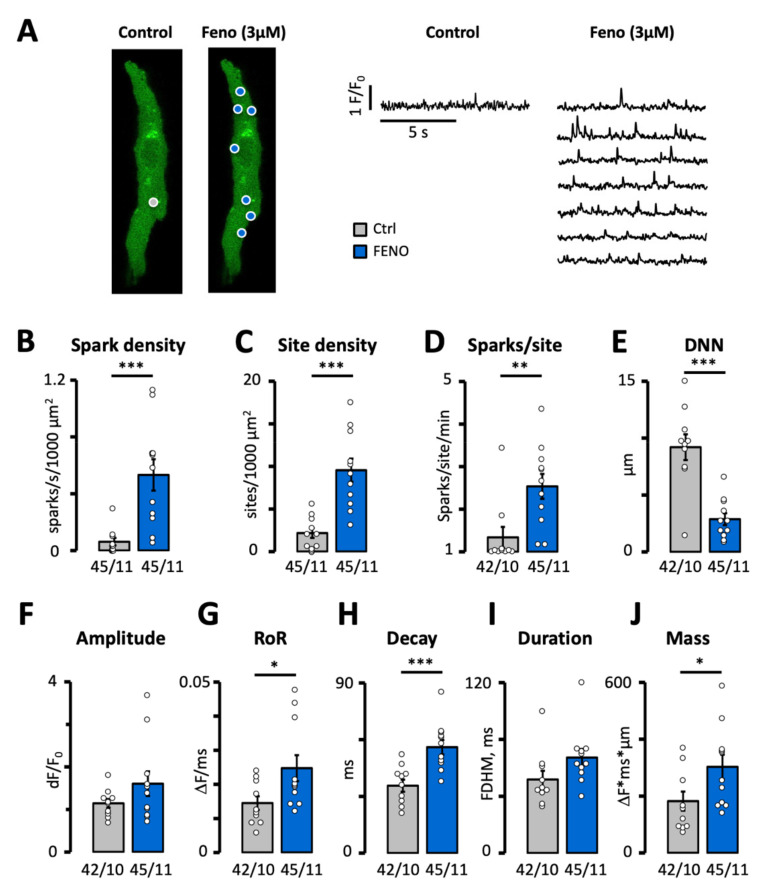
Effect of fenoterol on calcium spark frequency and properties. (**A**) Calcium spark recordings in a patient before and after exposure to 3 µM Feno. Spark sites are marked with circles and the signal for each site is shown on the right. Impact of Feno on (**B**) spark density, (**C**) spark site density, (**D**) spark frequency per site. *p*-values in B–D were obtained using Wilcoxon rank sum exact test. (**E**) Distance to nearest neighbor (DNN). (**F**) Spark amplitude. (**G**) Rate of rise (RoR). (**H**) Decay time constant. (**I**) Full duration at half maximum (FDHM). (**J**) Spark mass. *p*-values in E–J were obtained using Student’s *t*-test. Statistically significant differences between pairs of bars are indicated with *: *p* < 0.05, **: *p* < 0.01; ***: *p* < 0.001. Circles in (**B**–**J**) correspond with the values of individual data points. Number of cells/number of patients is given below bars.

Since the vast majority of β-blockers used in clinical practice selectively target β1-receptors, we wanted to determine the impact of β2-receptor activation on spontaneous calcium release events in human atrial myocytes in order to assess their potential contribution to pathological alterations in the intracellular calcium homeostasis. For this purpose, we exposed myocytes to a saturating dose (3 µM) of the selective β2-receptor agonist Feno, a drug that has been associated with an increased mortality rate in asthma patients medicated with it [24,25]. As shown in Figure 1A,B, Feno increased the density of calcium sparks 8-fold. This increase was due to an increase in both the density of sparks sites (Figure 1A,C) and the frequency of sparks per site (Figure 1A,D). Consequently, the distance from a spark to its nearest neighbor also decreased (Figure 1E). As shown in Figure 1F–H, Feno did not affect the amplitude, but increased the rate of rise (RoR) and decay (tau) of the sparks. As a result, the spark mass increased 1.7-fold (Figure 1J). This, combined with the higher density and shorter distance between sparks is expected to facilitate their fusion into calcium waves or trigger spontaneous calcium transients.

**Figure 2:**
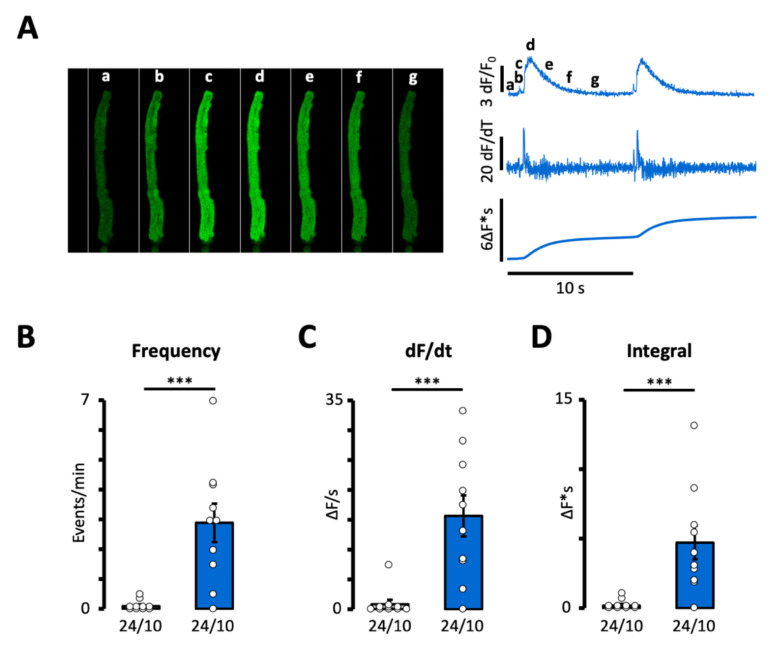
Effect of fenoterol on calcium wave frequency. (**A**) Recordings of a spontaneous calcium transient or wave before and after exposure to 3 µM Feno. Impact of Feno on (**B**) event frequency, (**C**) rate of rise of the calcium signal, (**D**) time integral of the calcium signal. *p*-values were obtained using Wilcoxon’s rank sum test. Statistically significant differences between pairs of bars are indicated with ***: *p* < 0.001. Circles in (**B**–**D**) correspond with the values of individual data points. Number of cells/number of patients is given below bars.

Accordingly, Figure 2A,B demonstrates that Feno increased the frequency of the calcium waves or spontaneous calcium transients 30-fold. In addition, Feno also dramatically increased both the rate of rise of the calcium signal (Figure 2C) and the time integral of the calcium wave or transient (Figure 2D), which, in turn, is expected to increase the amplitude of the resulting membrane depolarization.

In order to address the impact of β2-adrenergic stimulation relative to the activation of both β1- and β2-receptors, we compared the response to 3 µM Feno with the response to a saturating dose (100 nM) of Iso, a non-selective β-agonist. As shown in Table 2, the effect of Feno (measured in 45 cells from 11 patients) on the incidence and properties of calcium sparks and waves was similar to that of Iso (measured in 30 cells from 9 patients).

### 3.2. R-Carvedilol Dose-Dependently Reverses β2-Adrenergic Stimulation of Spontaneous Ca^2+^ Release

**Figure 3:**
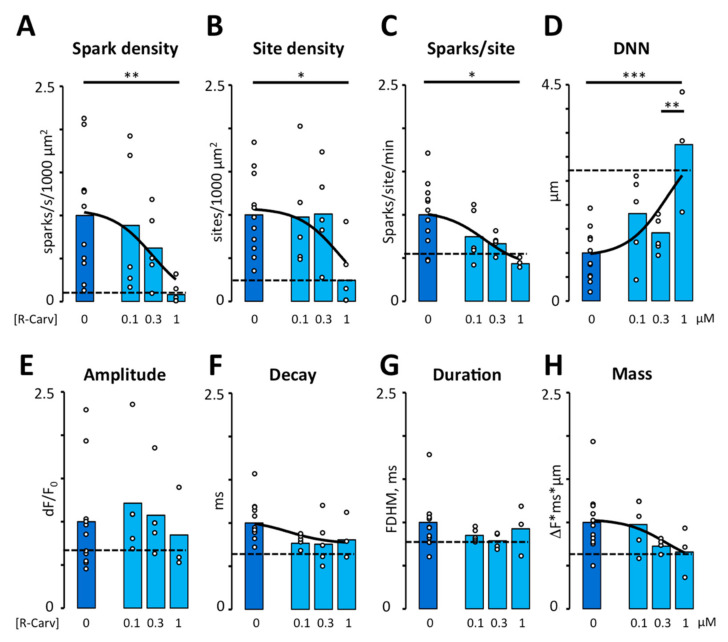
Effect of R-Carvedilol on calcium spark frequency and properties. Calcium spark frequency and properties were recorded in myocytes after addition of Feno and increasing doses of R-Carv (given below bars). (**A**) Spark density. (**B**) Spark site density. (**C**) Spark frequency per site. *p*-values in A–C were obtained using Kruskal–Wallis test followed by Bonferroni-adjusted multiple comparisons. (**D**) Distance to nearest neighbor (DNN). (**E**) Spark amplitude. (**F**) Decay time constant. (**G**) Full duration at half maximum (FDHM). (**H**) Spark mass. Data are from 45 cells/11 patients. *p*-values in D–H were obtained using ANOVA test followed by Tukey’s multiple comparisons test. Statistically significant differences between pairs of bars are indicated with *: *p* < 0.05, **: *p* < 0.01; ***: *p* < 0.001. Solid lines represent fitting using a Hill equation. Dashed lines represent mean values recorded in control conditions before exposure to Feno. Circles in (**A**–**H**) correspond with the values of individual data points.

Because R-Carv has been reported to selectively inhibit RyR2 activity without affecting β-adrenergic receptors [21], we explored the potential of R-Carv as a specific inhibitor of RyR2 activity. For this purpose, we first determined its ability to reverse the effects of β2-adrenergic stimulation with 3 µM Feno. As shown in Figure 3A,C, R-Carv reversed the stimulatory effects of Feno on calcium spark density and the numbers of sparks per site in a dose-dependent manner with IC50 values of 0.3 and 0.17 µM, respectively, reaching control levels at 1 µM R-Carv. The spark site density decreased abruptly at 1 µM. (Figure 3B). R-Carv also reversed the effects of Feno on the distance to the nearest spark (Figure 3D), spark amplitude, decay, and mass (Figure 3E,F,H) while spark duration was not significantly altered by Feno or R-Carv (Figure 3G). The IC50 values for the impact on calcium spark frequency and properties are summarized in Table 3.

**Figure 4:**
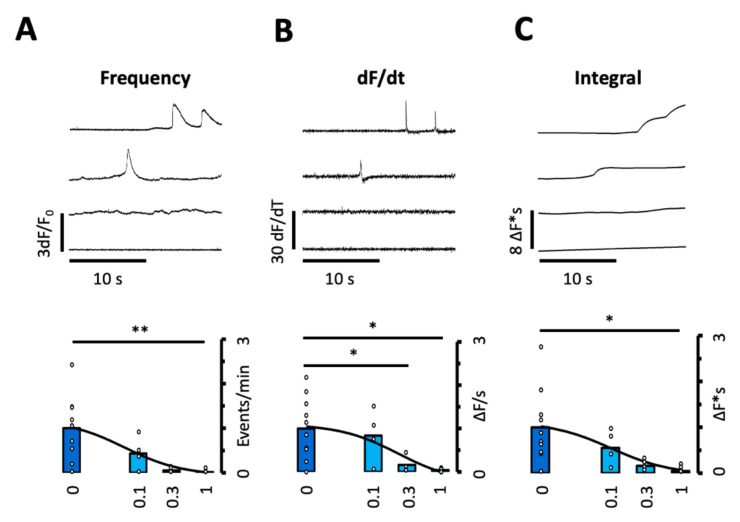
Effect of R-Carvedilol on calcium wave frequency and properties. The frequency and properties of spontaneous calcium transients or waves were recorded in myocytes after addition of Feno and increasing doses of R-Carv (given below bars). (**A**) Event frequency. (**B**) Rate of rise of the calcium signal. (**C**) Time integral of the calcium signal. Data are from 40 cells/10 patients. *p*-values were obtained using ANOVA test with Welch correction followed by Bonferroni-adjusted multiple comparisons. Statistically significant differences between pairs of bars are indicated with *: *p* < 0.05, **: *p* < 0.01. Solid lines represent fitting using a Hill equation. Circles in (**A**–**C**) correspond with the values of individual data points.

By reducing the number of sparks per site as well as their mass, R-Carv reduced the probability of large sparks coinciding in space and time and hence their propagation to neighboring clusters. Accordingly, R-Carv also reduced the frequency of spontaneous calcium transients and waves (Figure 4A) as well as the rate of rise and the time integral of these events (Figure 4B,C). 

This suggests that R-Carv not only reduces the frequency of spontaneous calcium waves or transients but also the rate of calcium release and the amount of calcium released per event, which, in turn, is expected to diminish both the frequency and amplitude of the resulting membrane depolarizations. The IC50 for the inhibition of spontaneous calcium waves, their area, and rate of release are shown in Table 3.

### 3.3. Impact of R- and S-Carvedilol Enantiomers on β2-Adrenergic Stimulation of RyR Phosphorylation at Ser-2808

**Figure 5:**
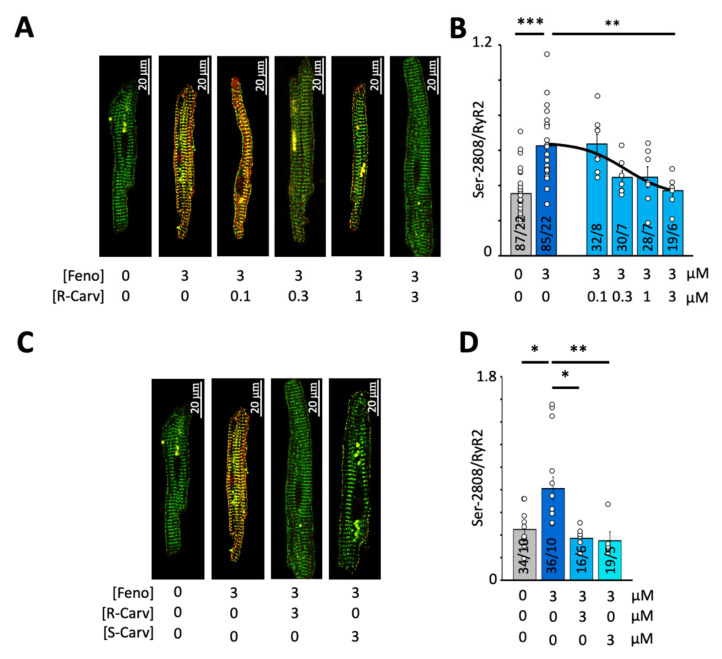
Effect of R- or S-Carvedilol on RyR2 phosphorylation at Ser-2808. (**A**) Overlay of total RyR2 (green) and Ser-2808-phosphorylated RyR2 (red) for different R-Carv concentrations (given below images). (**B**) Mean RyR2 phosphorylation at Ser-2808 determined as the fluorescence intensity ratio (Ser-2808/RyR2). (**C**) Overlay of total RyR2 (green) and Ser-2808-phosphorylated RyR2 (red) for 3 µM R- and S-Carv. (**D**) Mean RyR2 phosphorylation at Ser-2808 determined as the fluorescence intensity ratio (Ser-2808/RyR2). The number of cells/number of patients is given for each bar. Statistical significance was determined using a one-way ANOVA followed by Tukey’s multiple comparison test. Statistically significant differences between pairs of bars are indicated with *: *p* < 0.05, **: *p* < 0.01; ***: *p* < 0.001. Circles in (**B**,**D**) correspond with the values of individual data points. Number of patients is given for each bar.

Because R-Carv has been reported to selectively inhibit RyR2 activity without affecting β-adrenergic receptors [21], we analyzed its ability to reverse the RyR2 phosphorylation induced by β-adrenergic stimulation with 3 µM Feno. Figure 5A shows immunofluorescent labeling of total RyR2 and Ser-2808-phosphorylated RyR2 in control conditions, upon exposure to Feno and the subsequent exposure to different R-Carv concentrations. As shown in Figure 5B, R-Carv significantly and dose-dependently reduced Feno-induced RyR2 phosphorylation at Ser-2808 with an IC50 of 0.36 µM (see also Table 3). Moreover, comparison of the R- and S-Carv enantiomers showed that 1 µM of either of these abolished the stimulatory effect of 3 µM Feno on Ser-2808 phosphorylation (Figure 5C,D).

## 4. Discussion

### 4.1. Impact of β2-Adrenergic Stimulation on Intracellular Calcium Homeostasis in Human Atrial Myocytes 

β-adrenergic receptor blockers are, among others, used in clinical practice to treat patients with cardiac arrhythmia such as atrial fibrillation [26] and CPVT [27]. Among the β-adrenergic receptors, β1-receptors are reported as the predominantly expressed receptor accounting for 70%, while β2-receptors account for 30% [28,29]. Aligned with this finding, β-blockers commonly used in clinical practice, such as atenolol, bisoprolol, metoprolol, nebivolol, and betaxolol, selectively inhibit β1-receptors [30]. However, the activation of β1- and β2-receptors also depends on the concentration of circulating catecholamines and their binding constant to each receptor. Thus, noradrenaline and adrenaline binds stronger to β1- and β2-receptors, respectively [31]. Furthermore, β-adrenergic receptors are located in macromolecular clusters with a spatial distribution that is different for the β-receptors associated with the RyR2 and those associated with PLB and SERCA2a [32,33]. Together, this suggests that the relative contribution of β1- and β2-receptors may vary according to specific physiological and pathophysiological conditions. Indeed, the present findings demonstrate that selective activation of β2-receptors promotes both local (sparks) and global calcium release events (spontaneous calcium transients and waves) with an incidence that is comparable to that recorded when myocytes are activated with the non-selective β-agonist Iso. Moreover, Feno induced a significantly larger increase in the spark mass (estimated as the product of the spark amplitude, width, and duration) than Iso (see Table 2), facilitating the fusion of neighboring sparks and increasing the local membrane depolarization resulting from the extrusion of the released calcium by the Na-Ca exchanger. Thus, our finding suggests that non-selective or β2-selective β-blockers might be suitable to attenuate potentially arrhythmogenic spontaneous calcium release events under pathophysiological conditions that favor the activation of β2-adrenergic receptors. 

### 4.2. Ability of R- and S-Carvedilol Enantiomers to Reverse the Effects of β2-Adrenergic Stimulation on Ca^2+^ Homeostasis 

Since carvedilol is a non-selective β-blocker and the R-Carv enantiomer has been proposed to modify RyR2 opening without compromising β-adrenergic activity [18,20,21], we tested the ability of R-Carv to attenuate RyR2 phosphorylation at Ser-2808 and spontaneous calcium release in human atrial myocytes stimulated with 3 µM Feno. Under these conditions, we observed that R-Carv induced a dose-dependent reduction in calcium spark or wave frequency and also in RyR2 phosphorylation. Moreover, the IC50 for the inhibition of calcium sparks and waves was comparable to the IC50 for the inhibition of RyR2 phosphorylation, suggesting that the inhibitory effects of carvedilol could be due to reduced RyR2 phosphorylation caused by inhibition of the β-adrenergic receptors. In this regard, the Kd for R-Carv binding to β1- and β2-receptors is around 25 and 15 nM, respectively [13]. Thus, R-Carv at concentrations used in the present study would be expected to bind to a sizeable fraction of the β-receptors, inducing a dose-dependent reversal of the response to Feno that has a Kd for binding to β1- and β2-receptors near 9.1 and 0.93 µM, respectively [31]. Theoretically, the observed reduction in Ser-2808 phosphorylation could also be due to a direct inhibition of the RyR2 by R-Carv, which in turn could reduce CaMKII-dependent phosphorylation at Ser-2808. However, higher doses of R-Carv or non β-blocking carvedilol analogues are typically required to inhibit spontaneous calcium oscillations [14,34].

While we show here that both R- and S-Carv (3 µM) are able to fully reverse Feno-induced RyR2 phosphorylation at Ser-2808, we cannot rule out that S-Carv, which is expected to inhibit β1- and β2-receptors with a higher affinity, may be more efficient than R-Carv at lower concentrations. Functionally, this would reduce the IC50 for the racemic mixture of carvedilol with respect to the observed IC50 of 0.36 µM for R-Carv.

Interestingly, a high concentration of R-Carv (1 µM) induced an abrupt reduction in the spark site density and a rebound in spark duration, suggesting that carvedilol at this concentration might affect multiple mechanisms simultaneously. In this regard, β-adrenergic receptors are located in macromolecular clusters where the spatial distribution of those associated with the RyR2 is different from the distribution of those associated with PLB and SERCA2a [32,33]. Thus, R-Carv at concentrations lower than 1 µM might primarily regulate RyR2 gating but affect both RyR2 gating, PLB phosphorylation, SERCA activity, and SR calcium content at concentrations of 1 µM or higher.

Together, the present findings therefore suggest that carvedilol or R-Carv may be more suitable to treat cardiac arrhythmia than commonly used selective β1-blockers when physiological or pathophysiological conditions favor a differential activation of β2-receptors. In this regard, it is interesting to notice that a recent study has shown that treatment with β1-selective β-blockers are associated with a significantly higher risk for arrhythmic events in symptomatic children with CPVT than those treated with non-selective β-blockers. They therefore concluded that nadolol or other non-selective β-blockers should be preferred for treating symptomatic children with CPVT [35]. 

## 5. Conclusions

In summary, β2-adrenergic stimulation increases the incidence of calcium sparks and waves to levels observed with the non-specific β-adrenergic agonists, underscoring the relevance of β2-adrenergic receptors to the promotion of spontaneous calcium release in human atrial myocytes. Moreover, the non β-blocking R-Carv enantiomer can reverse these effects of Feno in a dose-dependent manner, settling a physiological basis for testing the ability of R-Carv to reverse arrhythmogenic calcium release in patients with atrial fibrillation or other cardiac arrhythmias associated with β2-adrenergic receptor activation.

## Figures and Tables

**Table 1:** Clinical Characteristics of the Study Population.

		(37 Patients)
	Age, Years	67.0 [65.0; 69.0]
	Sex (Female/Male)	16/21 (43.2%/56.8%)
Echocardiography	LAD index	2.35 [2.27; 2.43]
	LVEF, %	55.0 [53.0; 57.0]
Cardiovascular Risk Factors	No Smoking	16 (43.2%)
	Smoking	9 (24.3%)
	Ex smoking	11 (29.7%)
	Hypertension	21 (56.8%)
	Diabetes	8 (21.6%)
	Dyslipidemia	24 (64.9%)
Surgical Treatment	AVR	16 (43.2%)
	MVR	3 (8.1%)
	CABG	26 (70.3%)
Pharmacological Treatment	ACE inhibitor	14 (37.8%)
	ARB	6 (16.2%)
	Calcium antagonists	9 (24.3%)
	β-blockers	19 (51.4%)
	Acetylsalicylic acid	21 (56.8%)
	Statins	26 (70.3%)
	More than one treatment	27 (73.0%)

Categorical values are given as number of patients with the condition and % of patients in parenthesis. Continuous values are given as mean ± standard error. Smoking was divided into three groups (Non-, Ex- and smokers). LAD index, left atrial diameter index; LVEF, left ventricular ejection fraction; ACE inhibitor, angiotensin converting enzyme inhibitor; ARB, angiotensin receptor blocker; AVR, aortic valve replacement; MVR, mitral valve replacement; CABG, coronary artery bypass graft.

**Table 2:** Comparison of the Response to 3 µM Fenoterol and 100 nM Isoproterenol.

Properties	Fenoterol	Isoproterenol
CALCIUM SPARKS (cells/patients)	(45/11)	(30/9)
Density (events/s/1000 µm^2^)	0.52 ± 0.11	0.53 ± 0.12
Site density (sites/1000 µm^2^)	9.55 ± 1.36	11.5 ± 1.1
Sparks/site (events/s)	0.042 ± 0.005	0.039 ± 0.011
Distance to nearest neighbor (µm)	2.39 ± 0.39	3.66 ± 0.64
Amplitude (ΔF/F_0_)	1.48 ± 0.21	1.23 ± 0.16
Rate of Rise (ΔF/F_0_/s)	0.025 ± 0.004	0.028 ± 0.003
Tau (ms)	55.4 ± 3.8	43.3 ± 3.6 *
FDHM (ms) ^1^	65.7 ± 5.4	56.9 ± 2.8
FWHM (µm) ^2^	3.06 ± 0.16	2.44 ± 0.19 *
Mass (∆F × ms × µm)	289 ± 43	182 ± 27 *
CALCIUM WAVES ^3^		
Frequency (events/min)	2.89 ± 0.65	1.98 ± 0.94

^1^ Full duration at half maximum. ^2^ Full width at half maximum. ^3^ Includes both calcium waves and spontaneous calcium transients. * Statistical significance for differences between Feno and Iso is indicated with * *p* < 0.05.

**Table 3:** Dose-Dependent Effects of R-Carvedilol on Intracellular Calcium Homeostasis.

Properties	Maximum	Minimum	IC-50 ^1^
Spark density	1.06 ± 0.21	0 ± 0.67	0.30 ± 0.60
Site density	1.07 ± 0.16	0 ± 1.35	0.70 ± 1.95
Sparks/site (events/s)	1.03 ± 0.12	0.38 ± 0.29	0.17 ± 0.25
Distance to nearest neighbor (µm)	3.5 ± 1.62	0.99 ± 0.26	0.48 ± 0.71
Tau (ms)	1.0 ± 0.18	0.73 ± 0.14	0.06 ± 0.17
Mass (∆F × ms × µm)	1.03 ± 0.11	0.49 ± 0.52	0.37 ± 0.91
Wave frequency ^2^	1.18 ± 0.31	−0.09 ± 0.26	0.06 ± 0.08
dF/dt	1.08 ± 0.20	−0.25 ± 0.49	0.23 ± 0.31
Wave area	1.11 ± 0.27	−0.08 ± 0.34	0.10 ± 0.15
Ser-2808/RyR total	1.03 ± 0.06	0.54 ± 0.15	0.36 ± 0.41

^1^ Data were normalized to values recorded in the presence of Feno before addition of R-Carv, and fitted with a Hill equation using a rate of 1. Values are given as mean ± standard deviation. ^2^ Includes both calcium waves and spontaneous calcium transients.

## Data Availability

The data that support the findings of this study are available from the corresponding author upon reasonable request. Some data may not be made available because of privacy or ethical restrictions.
